# Protein-Based Fingerprint Analysis for the Identification of *Ranae Oviductus* Using RP-HPLC

**DOI:** 10.3390/molecules24091687

**Published:** 2019-04-30

**Authors:** Yuanshuai Gan, Yao Xiao, Shihan Wang, Hongye Guo, Min Liu, Zhihan Wang, Yongsheng Wang

**Affiliations:** 1 College of Pharmacy, Jilin University, Changchun 130021, China; ganys18@mails.jlu.edu.cn (Y.G.); xiaoyao17@mails.jlu.edu.cn (Y.X.); guohy18@mails.jlu.edu.cn (H.G.); liumin17@mails.jlu.edu.cn (M.L.); 2 College of Chinese Herbal Medicine, Jilin Agricultural University, Changchun 130118, China; wsh8805@163.com; 3 Department of Physical Sciences, Eastern New Mexico University, Portales, NM 88130, USA; zhihan.wang@enmu.edu

**Keywords:** *Ranae Oviductus*, identification, protein, RP-HPLC, fingerprint

## Abstract

This work demonstrated a method combining reversed-phase high-performance liquid chromatography (RP-HPLC) with chemometrics analysis to identify the authenticity of *Ranae Oviductus*. The fingerprint chromatograms of the *Ranae Oviductus* protein were established through an Agilent Zorbax 300SB-C8 column and diode array detection at 215 nm, using 0.085% TFA (*v*/*v*) in acetonitrile (A) and 0.1% TFA in ultrapure water (B) as mobile phase. The similarity was in the range of 0.779–0.980. The fingerprint chromatogram of *Ranae Oviductus* showed a significant difference with counterfeit products. Hierarchical clustering analysis (HCA) and principal component analysis (PCA) successfully identified *Ranae Oviductus* from the samples. These results indicated that the method established in this work was reliable.

## 1. Introduction

*Rana chensinensis* is mainly distributed in the Changbai Mountain area, China. *Ranae Oviductus* is the dried oviduct of female *Rana temporaria chensinensis* David. The *Ranae Oviductus* is a potent traditional Chinese medicine that has been used in clinical studies for thousands of years. Today it is widely used as a nutrient food. It has been reported that *Ranae Oviductus* has significant effects in enhancing immunity, anti-fatigue, anti-aging, and lowering blood fat [1,2,3,4]. As a precious traditional Chinese medicine, *Ranae Oviductus* has been in short supply because of its limited production [5]. Its high price and lucrative profits have tempted many counterfeit products, such as bullfrog oviduct, toad oviduct, or frog oviduct, to inundate the market, resulting in the uneven quality of *Ranae Oviductus* in the market [6,7]. Those counterfeits have a similar appearance but have less efficacy. To guarantee the quality of *Ranae Oviductus*, its authenticity identification has attracted more and more attention from the pharmacists, doctors, and medicinal scientists. The identification method of *Ranae Oviductus* is still under development. In the 2005 China Pharmacopoeia, the appearance and expansion degree were employed as discriminating items of *Ranae Oviductus* [8]. Our group has reported using UV spectra to identify *Ranae Oviductus* [9]. According to a previous study, it is difficult to identify the *Ranae Oviductus* and counterfeit products using traditional methods [10]. Therefore, it is essential to establish a highly reliable method for the identification of *Ranae Oviductus*.

More than 40% of the components in *Ranae Oviductus* are proteins and the proteins are the major bioactive components of *Ranae Oviductus* [11,12]. However, the identification of *Ranae Oviductus* and counterfeit products using HPLC based on protein has not been studied yet. In addition, reversed-phase high-performance liquid chromatography (RP-HPLC) is a simple, fast, and effective technique for protein separation and characterization, as used for protein in milk, wheat gliadin, and transgenic zein [13,14,15]. On the other hand, the fingerprint chromatogram is considered as a comprehensive qualitative and quantitative method for the identification of different species, especially in the quality assessment of traditional Chinese medicines [16]. The World Health Organization (WHO) has admitted the use of chromatographic fingerprints as an identification strategy for traditional Chinese medicinal preparations [17]. Many reports have employed HPLC fingerprint chromatograms to study the quality control of traditional Chinese medicines. For example, Lu et al. used the HPLC fingerprint to identify Chinese *Angelica* from related umbellifer herbs. Sun et al. analyzed polysaccharides from different *Ganoderma*. Li et al. established the fingerprint analysis of polyphenols, which were extracted from pomegranate peel, with reliable results [18,19,20].

In this work, the main proteins components of *Ranae Oviductus* were used as the study objects. We used RP-HPLC to establish a fingerprint method for the identification of *Ranae Oviductus*. Ten batches of *Ranae Oviductus* were collected from different main producing areas of the Changbai Mountains. A protein reference chromatogram was established using those *Ranae Oviductus*, based on protein composition similarity analysis. Furthermore, the difference between the authentic *Ranae Oviductus* and counterfeit products were investigated. The results were verified via a chemometric approach, utilizing principal component analysis and hierarchical clustering analysis. Both showed that the newly established *Ranae Oviductus* identification method was reliable.

## 2. Materials and Methods

### 2.1. Chemicals and Samples

The petroleum ether, guanidine hydrochloride, and ammonium sulfate analytical grade were purchased from Beijing Chemical Factory (Beijing, China). The dithiothreitol (DTT) and trifluoroacetic acid (TFA) were purchased from Sigma-Aldrich (St. Louis, MO, USA). The HPLC-grade acetonitrile (MeCN) and HPLC-grade methanol were purchased from Fisher (Fisher Scientific, USA). The ultrapure water was obtained from a gradient water purification system (Water Purifier, Sichuan, China).

**Figure 1:**
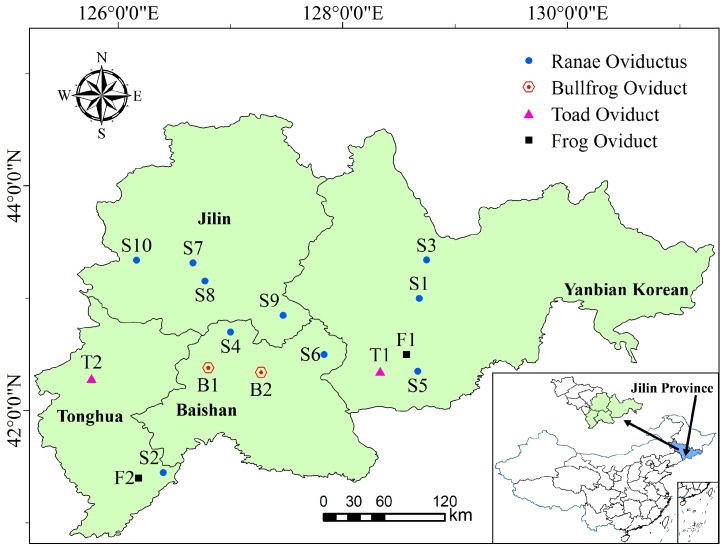
Distribution map of origins for *Ranae Oviductus* and its counterfeits in the Changbai mountain area.

*Ranae Oviductus*, bullfrog oviduct, toad oviduct and frog oviduct were provided by Jilin Province Rana Industry Association which were collected from the Changbai Mountain area in the Jilin province of China. The specific location is shown on the map in Figure 1. Ten batches of *Ranae Oviductus* samples were collected from different regions from the main producing area of the Changbai Mountain range. The specific collection information is shown in Table 1.

### 2.2. Protein Extraction

The dried *Ranae Oviductus* was pulverized into a powder (passing through a 20-mesh sieve) and degreased with petroleum ether at room temperature. After filtration, the powder was placed in an oven at 55 °C for 1 h. Afterward, 0.50 g of the sample was added to PBS buffer (50 mL, 0.1 M pH 7.4). After continuously stirring for 8 h, the mixture was centrifuged at 5000 r/min for 15 min. The supernatant was collected and the precipitate was extracted again. The two centrifugal supernatants were combined. To the supernatant, an ammonium sulfate solid was slowly added until to 60% saturation [21,22]. The mixture was centrifuged at 8000 r/min for 20 min after standing at 4 °C for 1 h. The precipitate was dissolved in 6 M guanidine hydrochloride (containing 10 mM DTT) [23,24], and dialyzed in distilled water in a dialysis bag (molecular weight cutoff: 8000 Da) for 12 h [25]. The sample solution was finally scaled to 5 mL with 6 M guanidine hydrochloride (containing 10 mM DTT) in a volumetric flask and filtrated with a 0.45 μm filter membrane prior HPLC injection [26]. The preparations of bullfrog oviduct, toad oviduct and frog oviduct were the same as that for *Ranae Oviductus*.

### 2.3. RP-HPLC Chromatography Analysis

The samples were separated using an Agilent Technologies 1200 Series liquid chromatograph (Agilent Technologies, Pittsburgh, PA, USA) equipped with a quaternary pump, autosampler, thermostatted column compartment, diode array detector (DAD), and UV detector. The columns used were the Agilent Zorbax 300SB-C8 column (250 × 4.6 mm, 5 μm) and Agilent Zorbax SB-C18 column (250 × 4.6 mm, 5 μm) with mobile phase A (0.085% TFA in *v/v* with acetonitrile) and mobile phase B (0.1% TFA in *v/v* with ultrapure water) [27,28]. Gradient elution was adopted as follows, from 12–30% A in the first 52 min, and from 30–44% A in the next 28 min. The injection volume was 20 μL. The optimized separation conditions were tested under the different detection wavelengths, flow rates and temperatures [29]. The data were recorded and processed using the Agilent Chemstation software.

### 2.4. Validation of the RP-HPLC Method

*Ranae Oviductus* sample (S1) was used to verify the RP-HPLC method. A precision analysis was carried out by repeatedly injecting the same solution 5 times on the same day. The repeatability was assessed by injecting 5 separate solutions obtained from the same *Ranae Oviductus* sample. The stability was evaluated by analyzing the same sample solution at different time periods of 0, 2, 4, 8, 16 and 24 h at room temperature.

### 2.5. Establishment of the HPLC Fingerprint

The common characteristic peaks and similarities of fingerprint data of 10 batches of *Ranae Oviductus* were investigated using the professional software Similarity Evaluation System for the Chromatographic Fingerprint, according to the recommendations of the State Food and Drug Administration (SFDA). The HPLC fingerprint data of the samples were imported to the evaluation system (the solvent peaks in the first 4 min were removed and the time window was set at 0.2 s). The calibration method was multi-point calibration. The significant common peaks were labeled as mark peaks and the reference chromatogram fingerprint was generated with a mean value method. The similarity of the fingerprint data was represented by a correlation coefficient (similarity) and the higher similarity between the two samples resulted in a correlation coefficient value close to 1. The correlation coefficients of all chromatograms of 10 batches of *Ranae Oviductus* samples were calculated throughout the study and a correlation analysis was performed.

### 2.6. Data Analysis

Hierarchical clustering analysis (HCA) is a cluster analysis technique that reflects the similarities and differences between samples in the form of a hierarchical tree diagram [30,31]. This method is easier to observe than the complex raw data. Based on the clustering method between different groups and the Pearson correlation intervals, SPSS (version 25.0; SPSS Inc., Chicago, IL, USA) was used to group the different samples in this study.

Principal component analysis (PCA) is a classification method that uses dimensionality reduction techniques to simplify numerous original variables into several representative composite indicators [32,33]. According to the contribution rate of each comprehensive indicator, the information of the original data could be reflected when using appropriate numbers of principal components (PCs) [34]. In this study, PCA was performed using SPSS (version 25.0; SPSS Inc., Chicago, IL, USA) and the fractional scatter plot was interpreted by the relationship between PC1, PC2, and PC3 for visual analysis of the data matrix.

## 3. Results and Discussion

### 3.1. Optimization of the RP-HPLC Conditions

**Figure 2:**
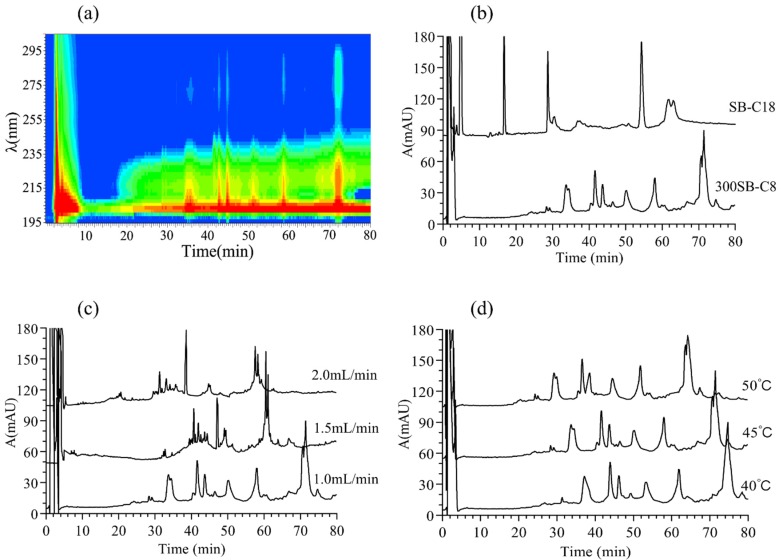
Optimization of reversed-phase high-performance liquid chromatography (RP-HPLC) separation method of the proteins from *Ranae Oviductus*. (**a**) The detection wavelength effect on the RP-HPLC chromatography of the *Ranae Oviductus* proteins. Diode array detector (DAD), 195–300 nm. (**b**) Column type effect on RP-HPLC chromatography of the *Ranae Oviductus* proteins (Agilent Zorbax 300SB-C8 column 250 × 4.6 mm, 5 μm, 300 Å and Agilent Zorbax SB-C18 column 250 × 4.6 mm, 5 μm, 80 Å). (**c**) Flow rate effect RP-HPLC chromatography of *Ranae Oviductus* (1.0 mL/min, 1.5 mL/min, 2.0 mL/min). (**d**) Temperature effect of RP-HPLC chromatography on the *Ranae Oviductus* proteins (40 °C, 45 °C, and 50 °C).

In order to improve the separation rate of the proteins in *Ranae Oviductus*, the *Ranae Oviductus* (S1) collected from the China Changbai mountain area were systematically investigated. The RP-HPLC chromatography method was optimized through the detection wavelength, separation column, flow rate and temperature. Three classical UV detection conditions were previously reported: 215 nm corresponding to the maximum absorption of peptide bonds; 254 nm corresponding to the maximum absorption of phenylalanine residues; and 280 nm corresponding to tyrosine and maximum absorption of tyrosine residues and tryptophan residues [35]. Figure 2a shows the UV absorption diagram of *Ranae Oviductus* using a diode array detector (DAD) with a wavelength range of 195–300 nm. The red region in the diagram indicated a larger absorption value. Although obvious solvent peaks around 215 nm were observed, the analysis of the core substance was not affected. The UV absorption diagram suggested that the separation effect at 215 nm was better than 254 nm and 280 nm.

Two types of columns (Agilent Zorbax SB-C18 column 250 × 4.6 mm, 5 μm, 80 Å and Agilent Zorbax 300SB-C8 column 250 × 4.6 mm, 5 μm, 300 Å) were used to examine the column effect on the protein separation of *Ranae Oviductus*. The results showed that the C8 column had a higher separation rate than the C18 column, which could be attributed to the large molecular weight of the proteins (Figure 2b). Therefore, the C8 column with a 300 Å pore diameter was selected for this study.

Since the flow rate of the mobile phase can affect the isolation efficiency, three flow rates (1.0, 1.5, 2.0 mL/min) were tested in this study. High flow rates showed that peaks overlapped (Figure 2c). The flow rate of 1.0 mL/min showed the highest separation effect and this, therefore, was chosen for the study.

On the other hand, the temperature played an important role in the RP-HPLC separation. Theoretically, high temperatures can increase the motion rate of proteins. In this study, three different temperatures (40, 45, and 50 °C) were investigated (Figure 2d). From the results, we could see that only one peak (t = 74.8 min) at 40 °C was observed, but two shoulder by shoulder peaks appeared at 45 and 50 °C. More proteins separated at 45 and 50 °C. Excessive temperature may damage the column’s sorbent, therefore, 45 °C was selected as the optimum temperature.

### 3.2. RP-HPLC Methodology Validation

The accuracy of the RP-HPLC method was investigated through consecutive tests five times, using the same sample solution (*Ranae Oviductus* sample S1) within one day. The relative standard deviations (RSD) of the retention times and peak areas of the 12 common peaks were smaller than 2.02% and 4.23%, respectively. The repeatability was determined by injecting five separate sample solutions of the *Ranae Oviductus* sample. The results showed that the RSD of the retention time and peak area of the 12 common peaks were smaller than 2.96% and 5.62%, which suggested that the RP-HPLC method had good repeatability. The stability test was carried out at room temperature for 0, 2, 4, 8, 16 and 24 h. The RSD of the retention times and peak area were smaller than 2.62% and 5.22%. All tests indicated that the RP-HPLC method established in this work satisfied the requirements of protein fingerprinting analysis of *Ranae Oviductus*.

### 3.3. HPLC Fingerprint of Ranae Oviductus Protein

**Figure 3:**
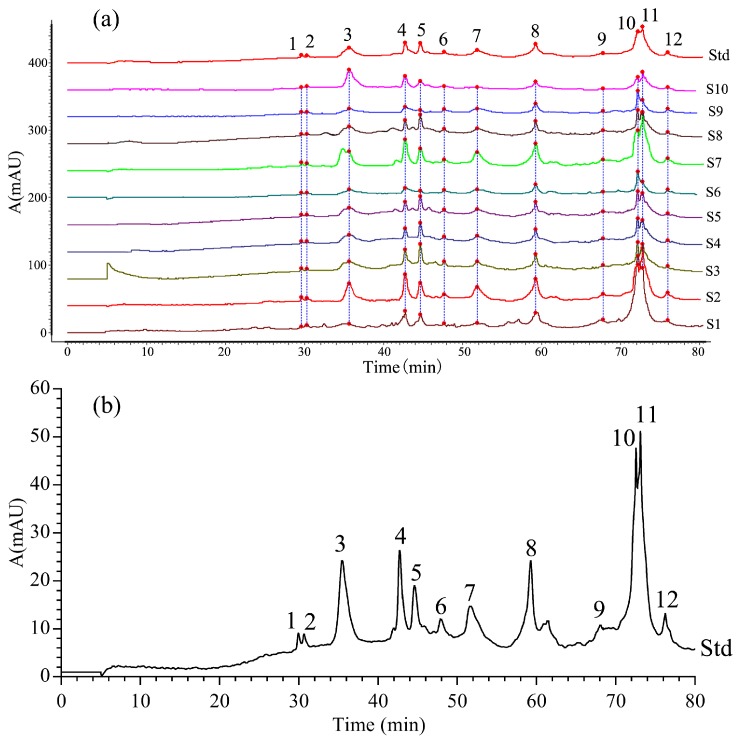
(**a**) HPLC fingerprint chromatographic spectra of 10 batches of *Ranae Oviductus* proteins. (**b**) The reference protein chromatographic spectra of *Ranae Oviductus*.

The protein chromatographic spectra of *Ranae Oviductus* collected from 10 sampling sites in Changbai Mountain area showed a similar profile using the optimized RP-HPLC method (Figure 3a). Based on the retention time, the 12 significant common-peaks were labeled with number 1 to 12. The 12 significant common-peaks in the *Ranae Oviductus* protein spectra were labeled as mark peaks according to the Chromatographic Fingerprint Similarity Evaluation System (2012 Edition) (Beijing, China). A reference fingerprint chromatographic spectrum of 10 batches of *Ranae Oviductus* was created (Figure 3b). The similarity was in the range of 0.779–0.980 (Table 2). The RSD value of the retention time of each common-peak was smaller than 4.70% and the RSD value of the relative peak area was smaller than 5.47%. This result pointed out that the common-peaks appearing in the chromatographic spectra were reliable in the analysis of *Ranae Oviductus*.

### 3.4. Fingerprint Spectra Analysis

**Figure 4:**
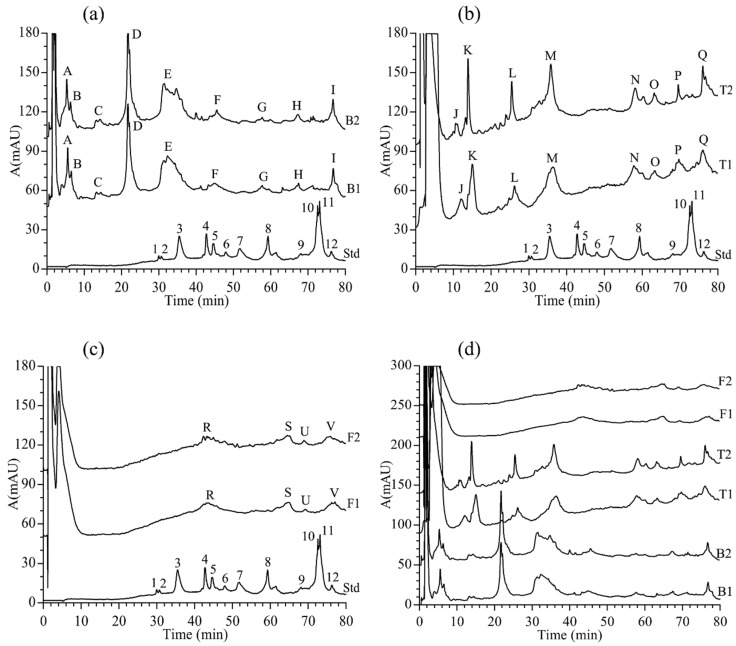
The comparison of *Ranae Oviductus* and counterfeit products. (**a**) Comparison of the protein HPLC fingerprint chromatogram of *Ranae Oviductus* (Std) and protein HPLC fingerprint chromatograms of the bullfrog oviduct (B1, B2). (**b**) Comparison of the protein HPLC fingerprint chromatogram of *Ranae Oviductus* (Std) and the protein HPLC fingerprint chromatograms of the toad oviduct (T1, T2). (**c**) Comparison of the protein HPLC fingerprint chromatogram of *Ranae Oviductus* (Std) and the protein HPLC fingerprint chromatograms of the frog oviduct (F1, F2). (**d**) Comparison of the protein HPLC fingerprint chromatograms of three counterfeits (bullfrog oviduct, toad oviduct, frog oviduct) of *Ranae Oviductus*.

The fingerprint spectra analysis of *Ranae Oviductus* and counterfeit products (bullfrog oviduct, toad oviduct and frog oviduct) were performed depending on the aforementioned optimized RP-HPLC method. The results showed a significant difference. By comparing Figure 4a,b, we could see that the significant common-peaks appeared at around 30 min in the reference fingerprint of *Ranae Oviductus*. In contrary, the counterfeit products, including the bullfrog oviduct, showed four common-peaks (peak A, peak B, peak C and peak D) in 0–30 min and the toad oviduct, showed three common-peaks (peak J, peak K, peak L) in the same time period. *Ranae Oviductus* showed 12 common-peaks (peak1-peak12) in 30–80 min, whereas, the bullfrog oviduct and toad oviduct only showed five common-peaks. The frog oviduct only showed four tiny common-peaks (Figure 4c), which was a finding consistent with a previous report. Huang, et al. [36] reported that the protein types in frog oviduct were less than that of other species by using the SDS-PAGE method. Both the protein extraction method and the RP-HPLC conditions were optimized according to the *Ranae Oviductus* sample, which may have not been adequate for frog oviduct. Through the comparison, we noticed that even the three counterfeit products had a significant difference (Figure 4d). The bullfrog oviduct (nine peaks) and toad oviduct (eight peaks) had more peaks than the frog oviduct (four peaks), but the retention time was different. Therefore, although *Rana chensinensis*, bullfrog, toad, and frog are similar amphibians, they are not the same species. Their genetic differences cause the expression of different types of proteins in the fallopian tubes, so that in RP-HPLC chromatographic spectra, they showed significant differences. Those differences can be used to identify *Ranae Oviductus* and counterfeit products.

### 3.5. Hierarchical Cluster Analysis (HCA)

**Figure 5:**
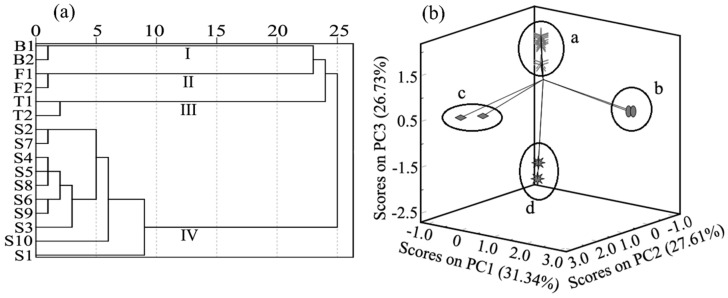
(**a**) The results of hierarchical cluster analysis of 10 batches of *Ranae Oveductus* and six counterfeit samples, (**b**) principal component analysis (PCA) score chart of 10 batches of *Ranae Oveductus* and six counterfeit samples in the first three principal components (PCs).

Hierarchical cluster analysis was carried out using the relative peak areas of the characteristic peaks of *Ranae Oviductus* and counterfeit products. The 16 samples were analyzed using SPSS 25.0 software and the results are shown in Figure 5a. Obviously, there were four clusters when the interval of abscissa was 10. Cluster I, Cluster II and Cluster III were composed of the bullfrog oviduct sample, frog oviduct sample and toad oviduct sample, respectively. Cluster IV referred to the 10 samples of *Ranae Oviductus* used in the establishment of the fingerprint. The sample S1 with low similarity to *Ranae Oviductus* also showed a low correlation in Cluster IV. When the interval of abscissa was 25, the sample was divided into two clusters, one authentic and another one counterfeit.

### 3.6. Principal Component Analysis(PCA)

As an effective data analysis technique, PCA has been used to study the classification of samples [37]. To directly reflect the difference between authentic and counterfeit products, 16 samples were used to perform the PCA analysis, based on the relative peak areas of the characteristic peaks of the samples. The variance contribution rates of the three main components (PC1, PC2, and PC3) were 31.34%, 27.61%, and 26.73%, respectively. The cumulative variance contribution rate of the three PCs was 85.68% and those variables reflected the majority of total information. To visualize the analysis results, the score charts were drawn using the three main components of PC1, PC2 and PC3 (Figure 5b). Four aggregation states are showed in Figure 5b. *Ranae Oviductus*, bullfrog oviduct, toad oviduct, and frog oviduct samples were classified in the a, b, c, and d regions, respectively. The *Ranae Oviductus* samples S1–10 could be classified in the same area (the a region), the bullfrog oviduct was classified in the b region, the toad oviduct was classified in the c region, and the frog oviduct was classified in the d region. The results were consistent with the HCA analysis, that both *Ranae Oviductus* and the counterfeit products were correctly classified. Comparing the similarity analysis with the HCA, PCA can provide a more visual comparison of the chromatograms.

## 4. Conclusions

This study used the RP-HPLC method and fingerprint technique to establish a chromatographic fingerprint of the proteins from *Ranae Oviductus*. Ten batches of *Ranae Oviductus* collected from the Changbai mountain area were used to analyze the protein components. The results showed 12 common-peaks in the reference fingerprint chromatographic spectrum. In combination with stoichiometry HCA and PCA, the results suggested that the method established in this work can satisfy the identification of *Ranae Oviductus* and counterfeit products. The method established in this work provides a promising approach for the identification of *Ranae Oviductus* and counterfeit products.

## Figures and Tables

**Table 1:** Origin and collecting date of the *Ranae Oviductus* samples and their counterfeits.

No.	Name of Medicine	Origin	Collection Date
S1	*Ranae Oviductus*	Yanbian, Jilin	2016.3
S2	*Ranae Oviductus*	Tonghua, Jilin	2016.1
S3	*Ranae Oviductus*	Yanbian, Jilin	2016.3
S4	*Ranae Oviductus*	Baishan, Jilin	2015.11
S5	*Ranae Oviductus*	Yanbian, Jilin	2016.3
S6	*Ranae Oviductus*	Baishan, Jilin	2015.11
S7	*Ranae Oviductus*	Jilin, Jilin	2016.12
S8	*Ranae Oviductus*	Jilin, Jilin	2016.12
S9	*Ranae Oviductus*	Jilin, Jilin	2015.11
S10	*Ranae Oviductus*	Jilin, Jilin	2015.11
B1	Bullfrog Oviduct	Baishan, Jilin	2016.12
B2	Bullfrog Oviduct	Baishan, Jilin	2016.12
T1	Toad Oviduct	Yanbian, Jilin	2016.10
T2	Toad Oviduct	Tonghua, Jilin	2016.11
F1	Frog Oviduct	Yanbian, Jilin	2016.10
F2	Frog Oviduct	Tonghua, Jilin	2016.11

**Table 2:** Similarity values of 10 batches of *Ranae Oviductus* protein and reference chromatographic fingerprint spectra.

No.	Similarity	No.	Similarity
S1	0.779	S6	0.976
S2	0.906	S7	0.884
S3	0.967	S8	0.877
S4	0.970	S9	0.980
S5	0.970	S10	0.861

## References

[1] Xie C., Zhang L.J., Zhang W.Y., Yang X., Fan L., Li X. (2010). Immunomodulatory effect of *Oviductus Ranae* on the mice. Chin. J. Gerontol..

[2] Li Z.G., Wang C. (2017). The anti-fatigue effect of protein hydrolysate of *Oviductus Ranae* on mice and its physiological mechanism. Chin. Sch. Phys. Educ..

[3] Mo Y., Yu M.J., Mo Y.L. (2011). Protective effect of *Oviductus Ranae* on D-galactose-induced aging mice. Chin. J. Gerontol..

[4] Peng F., Xu F., Liu B., Zhou B., Chen X., Zhao Q. (2003). Effects of *Rana Temporaria Chensinensis David* egg oil on blood lipid in hyperlipemia rats. Acad. J. Guangzhou Med. Coll..

[5] He Z., Tang X., Liu J., Yuan Y., Jiang C., Zhao Y., Wang Y. (2017). Application of rapid PCR to authenticate *Ranae Oviductus*. China J. Chin. Mater. Med..

[6] Hou G.L., Lu B.Z., Wang C.L. (2007). Authentic identification of *Ranae Oviductus*. Strait. Pharm. J..

[7] Jin P., Zhang Y., Wang H., Lan M., Zhang H., Sun J.M. (2018). Advances in identification of forest frog oil. Jilin J. Chin. Med..

[8] Liu J., Liu S., Liu C. (2009). True or false identification of oviductus ranae. Heilongjiang Med. Pharm..

[9] Wang Y.S., Jiang D.C., Bai X.X., Wang E.S. (2006). Identification Research *Rana temportva Chensinensis David*’s Quality with UASLG. Lishizhen Med. Mater. Med. Res..

[10] Zhang W., Wang W.N., Chen F.F., Zhang L., Yuan D. (2012). Quality evaluation of *Oviductus Ranae* and similar products and fakes. J. Shenyang Pharm. Univ..

[11] Hu X., Liu C.B., Chen X.P., Wang L.M. (2003). Main nourishment components of *Oviductus Ranae*. J. Jilin Agric. Univ..

[12] Hou Z.H., Zhao H., Yu B., Cui B. (2017). Comprehensively analysis of components in *Oviductus Ranae*. Sci. Technol. Food Ind..

[13] Ma L., Yang Y., Chen J., Wang J., Bu D. (2017). A rapid analytical method of major milk proteins by reversed-phase high-performance liquid chromatography. Anim. Sci. J..

[14] Han C., Lu X., Yu Z., Li X., Ma W., Yan Y. (2015). Rapid separation of seed gliadins by reversed-phase ultra performance liquid chromatography (RP-UPLC) and its application in wheat cultivar and germplasm identification. Biosci. Biotechnol. Biochem..

[15] Rodríguez-Nogales J.M., Cifuentes A., García M.C., Marina M.L. (2007). Characterization of Protein Fractions from Bt-Transgenic and Non-transgenic Maize Varieties Using Perfusion and Monolithic RP-HPLC. Maize Differentiation by Multivariate Analysis. J. Agric. Food Chem..

[16] Xie P., Chen S., Liang Y.-Z., Wang X., Tian R., Upton R. (2006). Chromatographic fingerprint analysis—a rational approach for quality assessment of traditional Chinese herbal medicine. J. Chromatogr. A.

[17] Sun J., Chen P. (2012). Chromatographic fingerprint analysis of yohimbe bark and related dietary supplements using UHPLC/UV/MS. J. Pharm. Biomed. Anal..

[18] Lu G.-H., Chan K., Liang Y.-Z., Leung K., Chan C.-L., Jiang Z.-H., Zhao Z.-Z. (2005). Development of high-performance liquid chromatographic fingerprints for distinguishing Chinese Angelica from related umbelliferae herbs. J. Chromatogr. A.

[19] Sun X., Wang H., Han X., Chen S., Zhu S., Dai J. (2014). Fingerprint analysis of polysaccharides from different Ganoderma by HPLC combined with chemometrics methods. Carbohydr. Polym..

[20] Zhu L., Fang L., Li Z., Xie X., Zhang L. (2019). A HPLC fingerprint study on Chaenomelis Fructus. BMC Chem..

[21] Harrysson H., Hayes M., Eimer F., Carlsson N.-G., Toth G.B., Undeland I. (2018). Production of protein extracts from Swedish red, green, and brown seaweeds, Porphyra umbilicalis Kützing, Ulva lactuca Linnaeus, and Saccharina latissima (Linnaeus) J. V. Lamouroux using three different methods. J. Appl. Phycol..

[22] Huang F., Cockrell D.C., Stephenson T.R., Noyes J.H., Sasser R.G. (1999). Isolation, purification, and characterization of pregnancy-specific protein B from elk and moose placenta. Biol. Reprod..

[23] Takakura D., Hashii N., Kawasaki N. (2014). An improved in-gel digestion method for efficient identification of protein and glycosylation analysis of glycoproteins using guanidine hydrochloride. Proteomics.

[24] Poulsen J.W., Madsen C.T., Young C., Poulsen F.M., Nielsen M.L. (2013). Using Guanidine-Hydrochloride for Fast and Efficient Protein Digestion and Single-step Affinity-purification Mass Spectrometry. J. Proteome Res..

[25] Mouecoucou J., Villaume C., Sanchez C., Mejean L. (2004). Effects of gum arabic, low methoxy pectin and xylan on in vitro digestibility of peanut protein. Food Res. Int..

[26] Vincent D., Rochfort S., Spangenberg G. (2019). Optimisation of Protein Extraction from Medicinal Cannabis Mature Buds for Bottom-Up Proteomics. Molecules.

[27] Ali I., Aboul-Enein H.Y., Singh P., Singh R., Sharma B. (2010). Separation of biological proteins by liquid chromatography. Saudi Pharm. J..

[28] Esteve C., Del Rio C., Marina M.L., Garcia M.C. (2011). Development of an ultra-high performance liquid chromatography analytical methodology for the profiling of olive (*Olea europaea* L.) pulp proteins. Anal. Chim. Acta.

[29] Gao P., Shi B., Li Z., Wang P., Yin C., Yin Y., Zan L. (2018). Establishment and Application of Infant Formula Fingerprints by RP-HPLC. Food Anal. Method..

[30] Cui L.L., Zhang Y.Y., Shao W., Gao D.M. (2016). Analysis of the HPLC fingerprint and QAMS from Pyrrosia species. Ind. Crop Prod..

[31] Kannel P.R., Lee S., Kanel S.R., Khan S.P. (2007). Chemometric application in classification and assessment of monitoring locations of an urban river system. Anal. Chim. Acta.

[32] Wang C., Zhang C.-X., Shao C.-F., Li C.-W., Liu S.-H., Peng X.-P., Xu Y.-Q. (2016). Chemical Fingerprint Analysis for the Quality Evaluation of Deepure Instant Pu-erh Tea by HPLC Combined with Chemometrics. Food Anal. Method..

[33] Goodarzi M., Russell P.J., Vander Heyden Y. (2013). Similarity analyses of chromatographic herbal fingerprints: A review. Anal. Chim. Acta.

[34] Nelson P.R.C., MacGregor J.F., Taylor P.A. (2006). The impact of missing measurements on PCA and PLS prediction and monitoring applications. Chemom. Intell. Lab.

[35] Esteve C., Del Río C., Marina M.L., García M.C. (2010). First Ultraperformance Liquid Chromatography Based Strategy for Profiling Intact Proteins in Complex Matrices: Application to the Evaluation of the Performance of Olive (*Olea europaea* L.) Stone Proteins for Cultivar Fingerprinting. J. Agric. Food Chem..

[36] Huang Y., Chang L., Zhang S.W., Yuan D. (2017). Electrophoresis methods for the characterizaiton of *Ranae Oviductus* and its adulterants. J. Shenyang Pharm. Univ..

[37] Fraige K., Pereira-Filho E.R., Carrilho E. (2014). Fingerprinting of anthocyanins from grapes produced in Brazil using HPLC–DAD–MS and exploratory analysis by principal component analysis. Food Chem..

